# Epidemiological trends of influenza A and B in one hospital in Chengdu and national surveillance data (2019–2024)

**DOI:** 10.3389/fcimb.2025.1603369

**Published:** 2025-06-12

**Authors:** Xiang Li, Chenlijie Yang, Lu Chen, Jian Ma, Zhongliang Hu

**Affiliations:** ^1^ Department of Laboratory Medicine, Sichuan Jinxin Xinan Women and Children Hospital, Chengdu, China; ^2^ College of Resources and Environment, Aba Teachers College, Wenchuan, China

**Keywords:** influenza A, influenza B, epidemiology, non-pharmaceutical interventions, COVID-19

## Abstract

**Background:**

Influenza A (Flu A) and Influenza B (Flu B) are major contributors to seasonal epidemics, causing significant morbidity and mortality worldwide. Understanding their epidemiological trends is essential for optimizing prevention and control strategies.

**Objective:**

This study aims to analyze the epidemiological trends of Flu A and Flu B, compare hospital-based and national surveillance data, and evaluate the impact of COVID-19 on influenza transmission to provide scientific evidence for influenza control measures.

**Methods:**

We analyzed influenza positivity rates from Sichuan Jinxin Xinan Women and Children Hospital data (HD) and Chinese National Influenza Center (CNIC) between 2019 and 2024. Temporal trends, subtype distributions, and the effects of non-pharmaceutical interventions (NPIs) were assessed.

**Results:**

Influenza activity exhibited significant temporal variations. In HD, the highest cumulative positivity rate of Flu A + Flu B was observed in 2023 (31.9%), whereas the lowest rate occurred during the COVID-19 pandemic (2020–2022), with a nadir in 2021 (2.0%). Flu A remained the predominant subtype in HD except in 2021, whereas CNIC data showed a relatively higher proportion of Flu B. Weekly positivity rates displayed distinct seasonal trends in CNIC data but not in HD. A comparative analysis of pre-pandemic (2019), pandemic (2020–2022), and post-pandemic (2023–2024) phases indicated that NPIs had a stronger suppressive effect on Flu A than on Flu B.

**Conclusion:**

Hospital-based and national influenza surveillance data showed heterogeneity in subtype proportions, seasonal trends, and pandemic-related impacts. These findings underscore the importance of integrating multiple surveillance sources for a comprehensive understanding of influenza dynamics. Enhancing vaccine coverage, implementing targeted public health interventions, and optimizing resource allocation are crucial for mitigating the influenza burden in the post-pandemic era.

## Introduction

Influenza, commonly known as the flu, is an acute viral respiratory disease caused by infection with influenza viruses, primarily seasonal influenza A (Flu A) and B (Flu B) viruses (Uyeki, 2021; Bi et al., 2024). These viruses circulate globally, leading to seasonal epidemics that significantly impact public health (Uyeki et al., 2022). Hospitalization rates are particularly elevated among vulnerable populations, including children, the elderly, and individuals with underlying health conditions (Thomas, 2023). The elderly population, particularly those aged 65 and older, experiences the highest mortality rates (approximately 90%) due to influenza (Mi et al., 2025). It is estimated that seasonal influenza causes between 290,000 and 650,000 deaths worldwide each year (Cozza et al., 2021). Studying the epidemiological trends of influenza can provide valuable insights for future prevention and control measures.

In China, influenza remains a significant public health concern. The epidemiological trends of Flu A and Flu B in the Chinese population exhibit a certain pattern. Overall, Flu A infection rates are generally higher than those of Flu B, with Flu A peaking during the winter and early spring months (December to March of the following year). In contrast, Flu B tends to peak later, sometimes emerging at the end of winter or in spring (Huang W-J. et al., 2022; Lei et al., 2022; Xie et al., 2024; Qu et al., 2025). In recent years, the COVID-19 pandemic has influenced influenza transmission patterns. Non-pharmaceutical interventions (e.g., mask-wearing, social distancing) led to a decline in influenza activity in certain years (Feng et al., 2021; Huang Q-M. et al., 2022). However, as control measures eased, Flu A and Flu B circulation gradually rebounded (Zhang et al., 2024; Cowling et al., 2020).

Existing studies may lack long-term surveillance data for specific regions (Hammond et al., 2022; Soudani et al., 2022; Langer et al., 2023). While the National Influenza Center provides nationally representative data, it does not include detailed analyses of case positivity rates in regional healthcare facilities. Additionally, influenza transmission patterns may have been disrupted during the COVID-19 pandemic and subsequent recovery period, yet research on this impact remains limited.

Here we analyzed the trends in Flu A and Flu B positivity rates at Sichuan Jinxin Xinan Women and Children Hospital from 2019 to 2024 and compared them with data from the Chinese National Influenza Center (CNIC). Our findings revealed notable heterogeneity between hospital data (HD) and CNIC influenza surveillance data in terms of influenza subtype proportions, seasonal fluctuations, and the impact of COVID-19, highlighting the potential limitations of relying on a single data source for assessing influenza dynamics. Additionally, we examined influenza trends across the pre-pandemic, pandemic, and post-pandemic periods and found that non-pharmaceutical interventions (NPIs) during COVID-19 had a stronger suppressive effect on Flu A than on Flu B. These findings underscore the need for enhanced influenza vaccine coverage, precise surveillance, and adaptive resource allocation in the post-pandemic era, particularly in response to Flu B’s seasonal peaks and the risk of mixed influenza outbreaks.

## Materials and methods

### HD data collection

#### Virus detection reagents and instruments

FluA and FluB antigens were detected using a colloidal gold-based influenza antigen detection kit (Product Registration Number: National Medical Device Registration Certificate Number: 20143401922) manufactured by InTec (https://www.asintec.com/product/30). This rapid immunochromatographic assay utilizes a double-antibody sandwich principle. The test strip is pre-coated with monoclonal antibodies targeting the nucleoproteins of FluA and FluB at detection zones A and B, respectively, and with goat anti-mouse IgG antibodies in the control zone (C). If viral antigens are present in the sample, they bind to colloidal gold-labeled antibodies, forming antigen-antibody complexes that migrate along the nitrocellulose membrane and produce visible color bands, allowing for qualitative detection of FluA and FluB.

#### Sample collection and processing

*Sample type*: Throat swabs were collected from patients with influenza-like illness (ILI) at Sichuan Jinxin Xinan Women and Children Hospital from 2019 to 2024. The swabs were gently rubbed against the posterior pharyngeal wall and bilateral tonsils, rotated, and held in place for 10 seconds to ensure sufficient sample collection.

*Sample preservation*: Immediately after collection, swabs were immersed in the provided lysis buffer (0.01 M phosphate-buffered solution, pH 7.2 ± 0.2), stirred thoroughly 10 times, and squeezed against the tube wall to release the antigens. Samples were tested within 2 hours of collection to avoid degradation, and repeated freeze-thaw cycles were avoided.

#### Detection procedure

*Reagent preparation*: Prior to testing, unopened reagent kits were equilibrated to room temperature (15–30°C). Once opened, test cassettes were used within 1 hour to prevent moisture interference.

*Sample application*:

A micropipette was used to transfer 80 μL (approximately 2–3 drops) of the lysed sample solution into the sample well of the test cassette.

A timer was started, and the test was incubated at room temperature for 10–15 minutes before result interpretation. Results read beyond this time frame were considered invalid.

*Result interpretation:*

Positive: A red band appeared in detection zone A (FluA) and/or B (FluB), with a visible control band (C).

Negative: Only the control band (C) appeared, with no visible bands in zones A or B.

Invalid: No control band (C) appeared, indicating a failed test requiring retesting.

This study was approved by the Ethics Committee of Sichuan Jinxin Xinan Women and Children Hospital.

### CNIC data collection

Influenza surveillance data were obtained from CNIC, which systematically collects and reports influenza activity across China. The dataset includes weekly influenza positivity rates and subtype distributions from sentinel hospitals and laboratories nationwide. For this study, we extracted relevant data from the CNIC database covering the period from 2019 to 2024. Data were accessed through official CNIC (https://ivdc.chinacdc.cn/cnic/zyzx/lgzb/202411/t20241115_302662.htm).

### Statistical analysis

Data were analyzed using GraphPad Prism 9.0. Group differences were assessed using the Kruskal-Wallis test followed by Dunn’s multiple comparison test. Bar graphs were presented as mean ± SD and generated using GraphPad Prism 9.0. Polar coordinate plots were created by https://www.bioinformatics.com.cn, an online platform for data analysis and visualization. A p-value of <0.05 was considered statistically significant.

## Results

### Influenza positivity rates in HD and CNIC from 2019 to 2024

**Figure 1:**
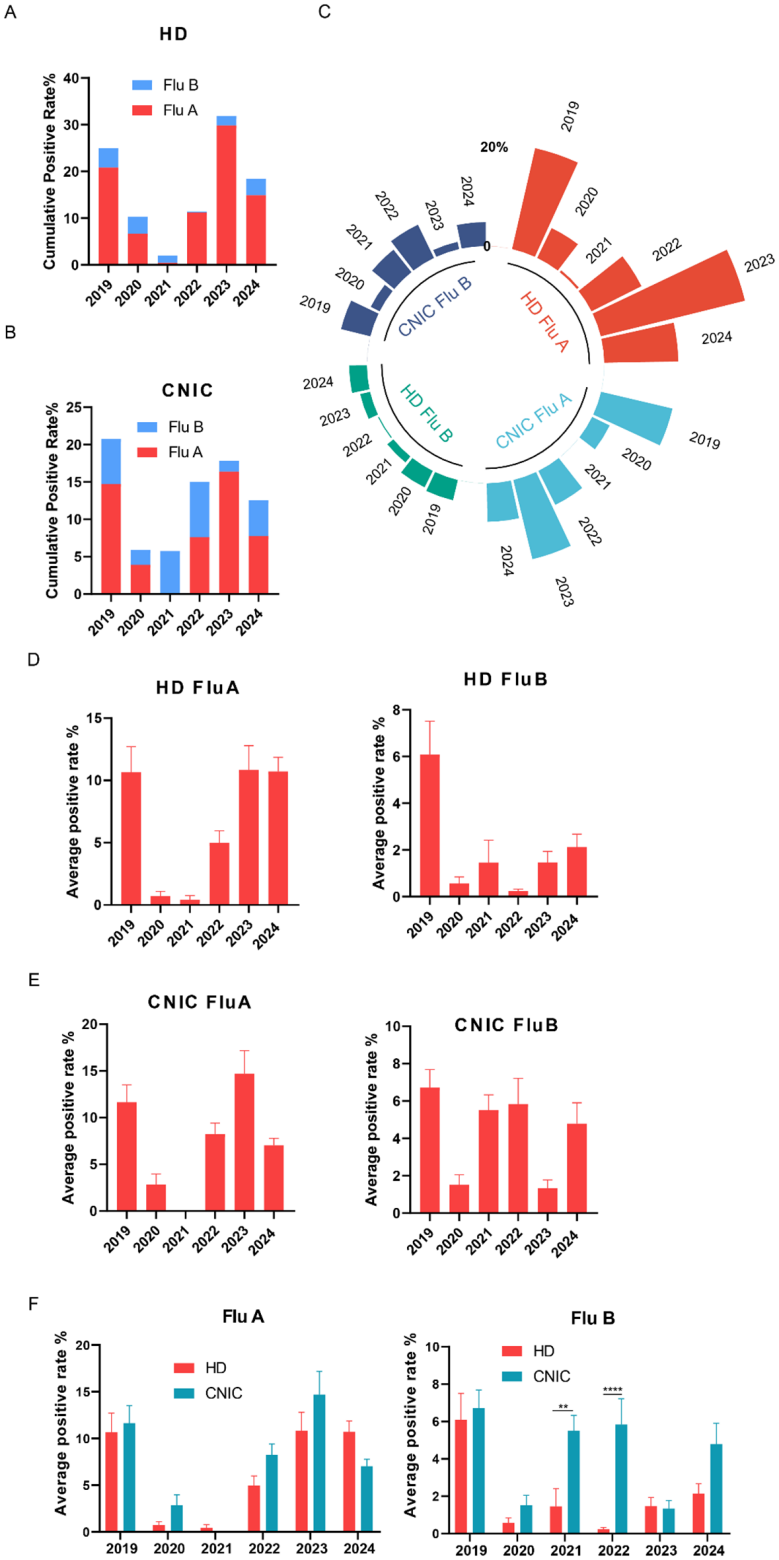
Analysis of influenza A and B positivity rates in HD and CNIC datasets. **(A)** Cumulative positivity rates of influenza A and B in HD. This figure illustrates the cumulative positivity rates of influenza A (Flu A) and influenza B (Flu B) cases in the HD (hospital data) over the study period. **(B)** Cumulative positivity rates of Flu A and Flu B in CNIC data. This figure illustrates the cumulative positivity rates of Flu A and Flu B cases in the CNIC (Chinese National Influenza Center) dataset over the study period. **(C)** Polar plot of Flu A and Flu B positivity rates in HD and CNIC data from 2019 to 2024. This figure presents a polar plot comparing the positivity rates of Flu A and Flu B in both HD and CNIC datasets over the period from 2019 to 2024. **(D)** Bar chart of average weekly positivity rates for Flu A and Flu B in HD data. This figure presents a bar chart depicting the average weekly positivity rates of Flu A and Flu B cases within the HD. **(E)** Bar chart of average weekly positivity rates for Flu A and Flu B in CNIC data. This figure presents a bar chart depicting the average weekly positivity rates of Flu A and Flu B cases within the CNIC dataset. **(F)** Comparison of average positivity rates of Flu A and Flu B between HD and CNIC datasets, shown as paired bars by year. ** indicates p<0.01; ****indicates p<0.0001.

We analyzed the positivity rates of Flu A and Flu B in HD from 2019 to 2024 and found that the highest cumulative positivity rate of Flu A + Flu B was observed in 2023 (31.9%), followed by 2019 (25.0%) and 2024 (18.4%). In contrast, the cumulative positivity rates were recorded during the COVID-19 pandemic period, with 2020 at 10.3%, 2021 at 2.0%, and 2022 at 11.4%, indicating a substantial decline during the peak pandemic years and a gradual rebound thereafter. Flu A was the predominant subtype in the cumulative positivity rates (**Figures 1A, C**). Additionally, an analysis of influenza surveillance data from CNIC during the same period revealed a slightly different trend. The cumulative positivity rates of Flu A + Flu B, ranked from highest to lowest, were as follows: 2019 (20.8%), 2023 (17.8%), 2022 (15.0%), 2024 (12.5%), 2020 (5.9%), and 2021 (5.8%). Notably, the proportion of Flu B appeared to be slightly higher in the CNIC dataset compared to HD (**Figures 1B, C**). Further analysis of the weekly average positivity rates in HD revealed that Flu A maintained an average of approximately 10% in 2019, 2023, and 2024, while in 2020 and 2021, the average dropped to around 1%, with a moderate increase observed in 2022 (approximately 5%). For Flu B, the average positivity rate was 6.1% in 2019, with significantly lower rates (*p*<0.05) observed in the subsequent years (**Figure 1D**). Analysis of CNIC data revealed that the weekly average positivity rate for Flu A peaked in 2023 at 14.7%, followed by 11.6% in 2019, with the lowest rate observed in 2021 (<0.1%). For Flu B, lower average positivity rates were noted in 2020 (1.5%) and 2023 (1.3%), while in other years, the rates exceeded 4% (**Figure 1E**). In addition, we compared the average positivity rates of Flu A and Flu B between the HD and CNIC datasets from 2019 to 2024. The results showed that there were no statistically significant differences in the average positivity rates of Flu A between HD and CNIC. For Flu B, however, the positivity rates in CNIC were significantly higher than those in HD in both 2021 (*p*<0.01) and 2022 (*p*<0.0001) (**Figure 1F**).

### Proportional analysis of influenza A and B positivity rates in HD and CNIC datasets

**Figure 2:**
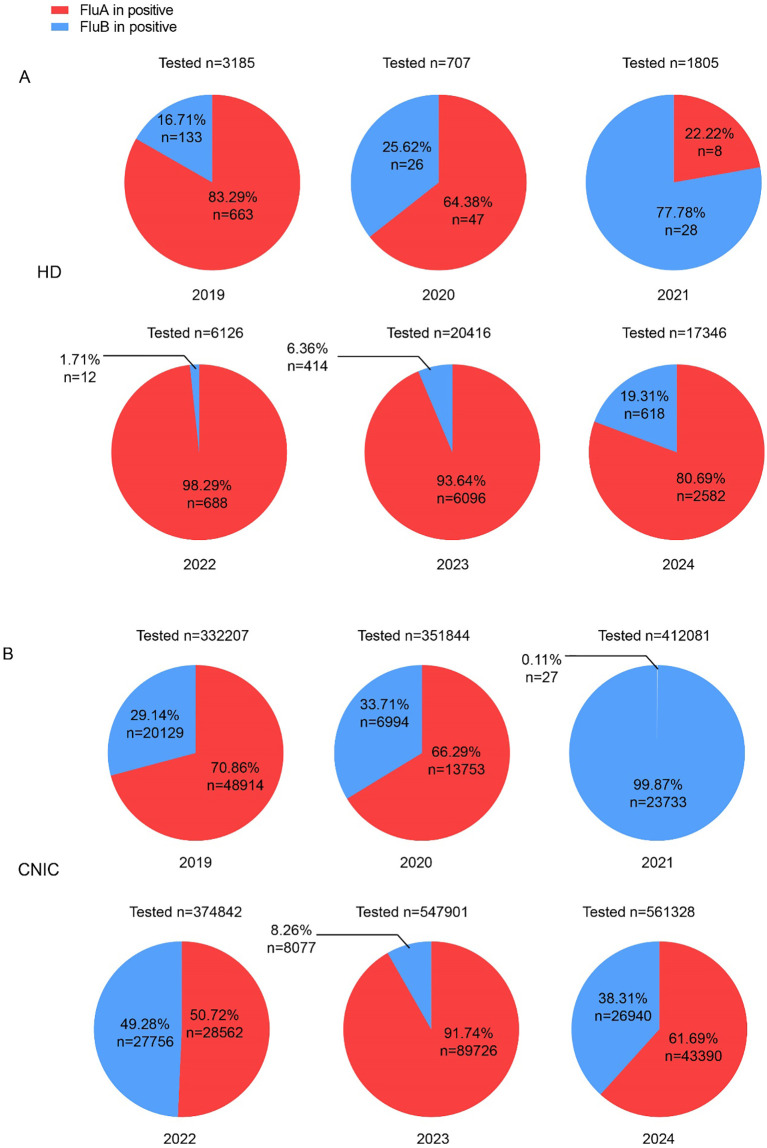
Proportions of influenza A and B cases among influenza-positive cases in HD and CNIC datasets. **(A)** Pie chart depicting the proportion of influenza A and B cases among influenza-positive cases in HD. **(B)** Pie chart depicting the proportion of influenza A and B cases among influenza-positive cases in CNIC data. HD, hospital data; CNIC, Chinese National Influenza Center.

Analysis of influenza data from both HD and CNIC revealed notable differences in the positivity rates of Flu A and Flu B. To further investigate the relationship between these subtypes among positive cases, we examined their respective proportions. In the HD dataset, Flu A predominated in all years except 2021, with its proportion exceeding 80% in 2019, 2022, 2023, and 2024 (**Figure 2A**). Conversely, in the CNIC dataset, only in 2023 did Flu A’s proportion surpass 80%. Notably, in 2021, Flu B accounted for an overwhelming 99.87% of positive cases (**Figure 2B**).

### Weekly trends in influenza A and B positivity rates in HD and CNIC datasets (2019–2024)

**Figure 3:**
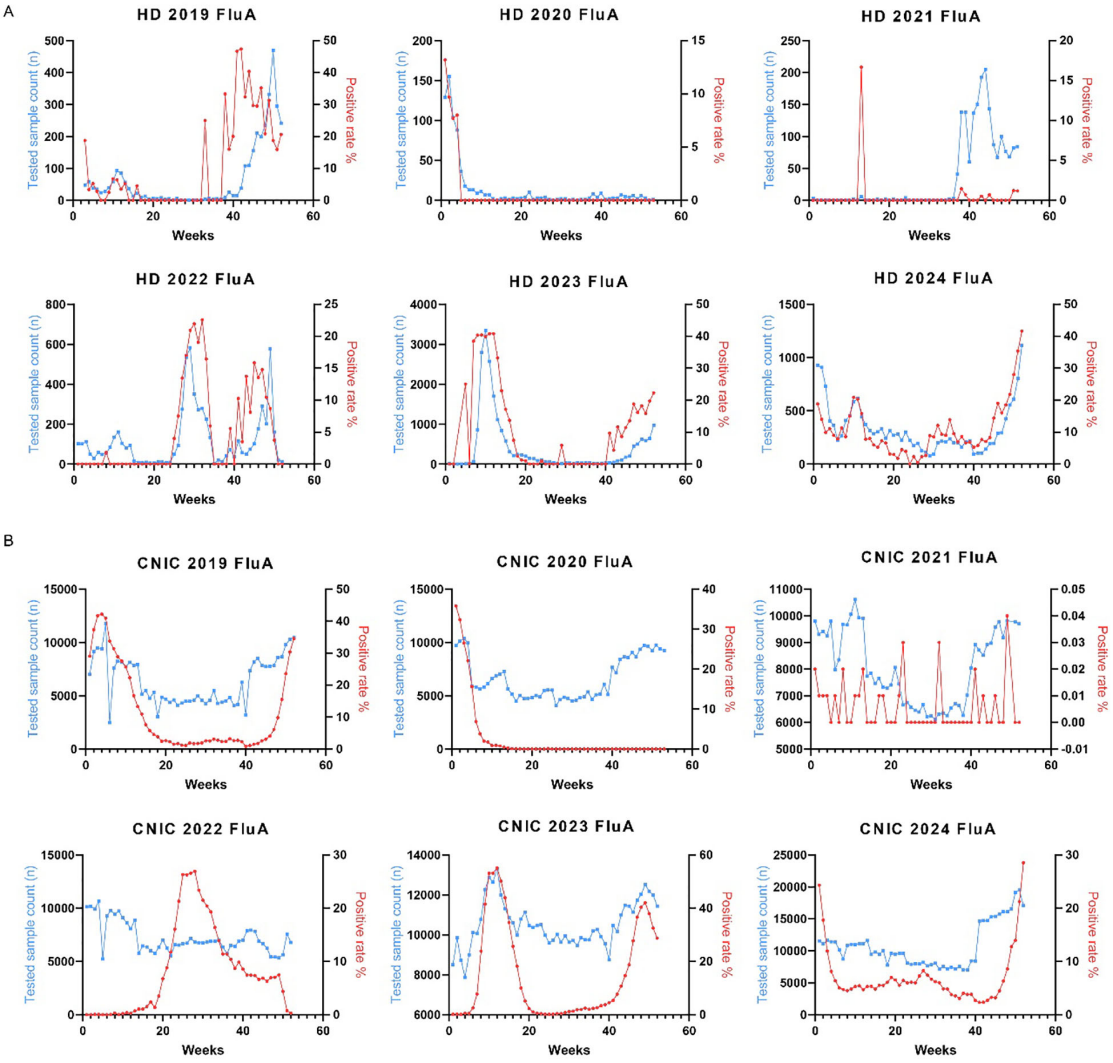
Weekly distribution of influenza A testing samples and positivity rates in HD and CNIC datasets. **(A)** Weekly distribution of influenza A testing samples and positivity rates in HD. **(B)** Weekly distribution of influenza A testing samples and positivity rates in CNIC. HD, hospital data; CNIC, Chinese National Influenza Center.

To investigate the annual trends in Flu A positivity rates, we analyzed their weekly variations from 2019 to 2024. In the HD dataset, no distinct seasonal patterns were observed in Flu A positivity rates during this period (**Figure 3A**). Conversely, the CNIC data exhibited more defined seasonal trends, with notable peaks occurring during the first 15 weeks and the last 10 weeks of 2019, 2023, and 2024 (**Figure 3B**).

**Figure 4:**
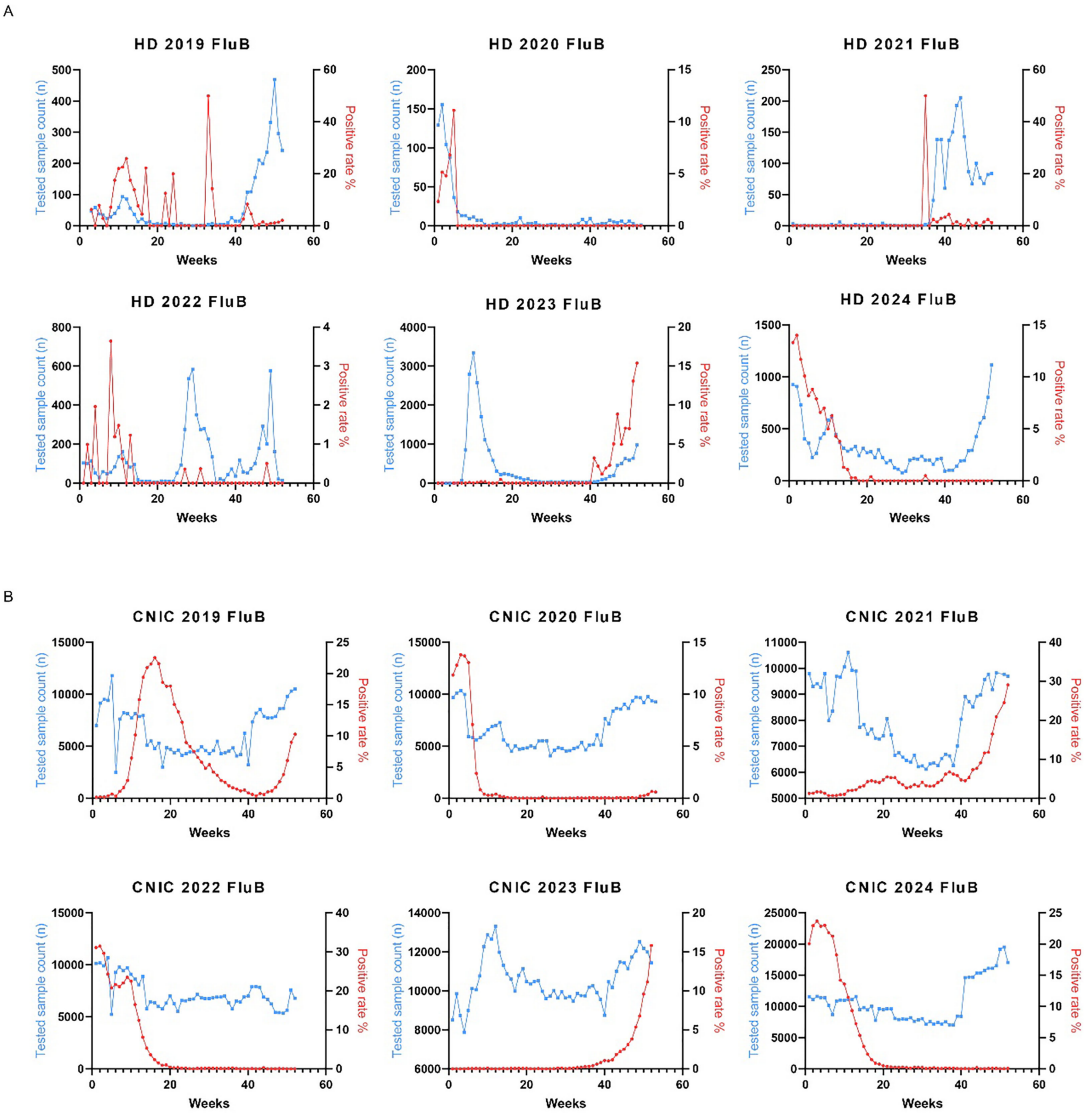
Weekly distribution of influenza B testing samples and positivity rates in HD and CNIC datasets. **(A)** Weekly distribution of influenza B testing samples and positivity rates in HD. **(B)** Weekly distribution of influenza B testing samples and positivity rates in CNIC. HD, hospital data; CNIC, Chinese National Influenza Center.

Similarly, we analyzed the weekly variations in Flu B positivity rates across different years. In the HD dataset from 2022 to 2024, Flu B positivity rates peaked during the initial 10 weeks and the final 10 weeks of each year (**Figure 4A**). The CNIC data exhibited a comparable pattern, with significant peaks in Flu B positivity rates occurring within the first and last 10 weeks of the year (**Figure 4B**).

### Impact of the COVID-19 pandemic on influenza positivity rates in HD and CNIC datasets

**Figure 5:**
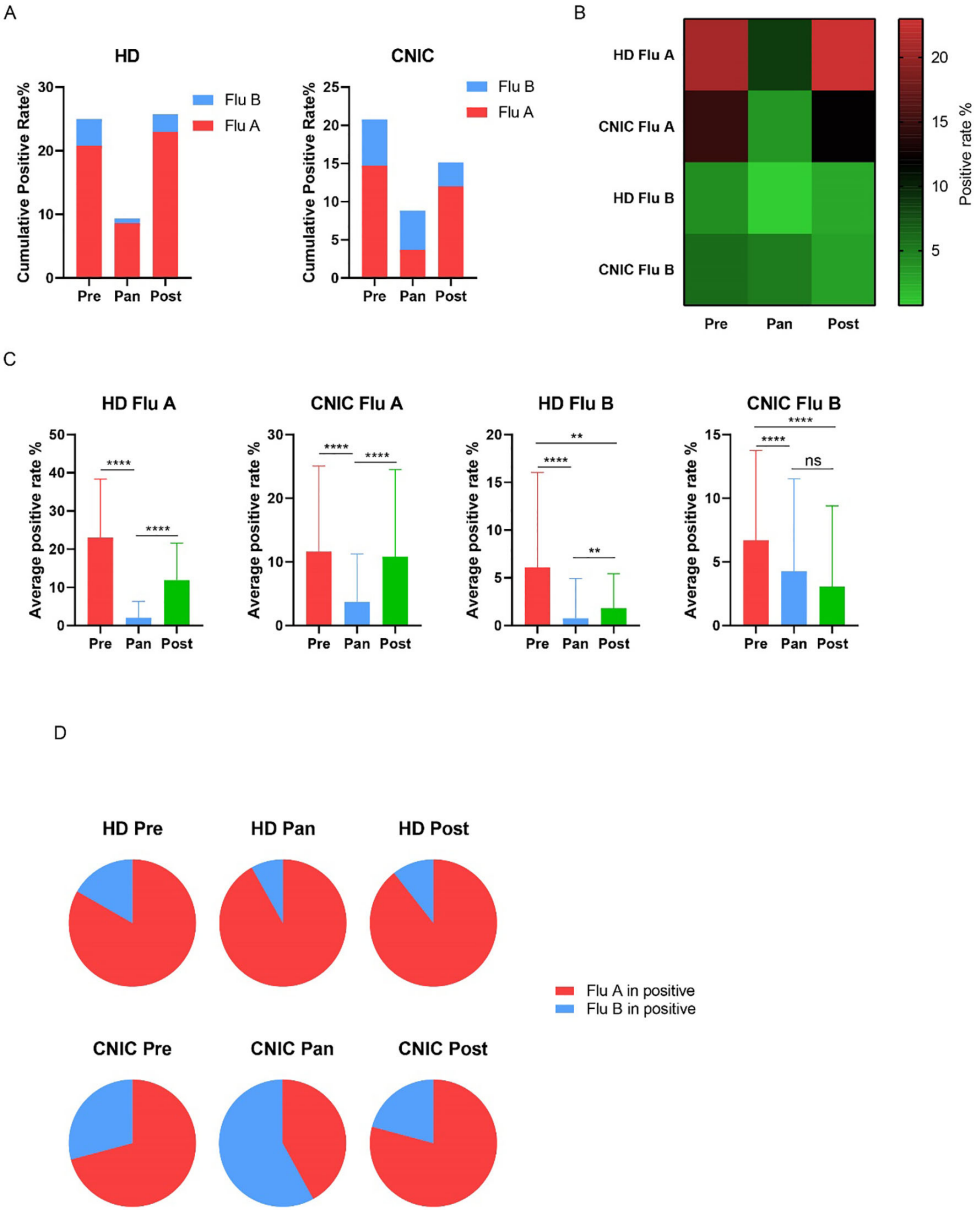
Analysis of influenza A and B positivity rates in HD and CNIC datasets during the Pre, Pan, and Post COVID-19 periods. **(A)** Bar charts depict the cumulative positivity rates of influenza A and B in HD and CNIC datasets during the pre-pandemic (Pre), pandemic (Pan), and post-pandemic (Post) COVID-19 periods. **(B)** Heatmap depicting the positivity rates of influenza A and B in HD and CNIC datasets during the Pre, Pan, and Post COVID-19 periods. **(C)** Bar charts depicting the weekly average positivity rates of influenza A and B in HD and CNIC datasets during the P Pre, Pan, and Post COVID-19 periods. **(D)** Pie charts depicting the proportions of influenza A and B cases among influenza-positive cases in HD and CNIC datasets during the Pre, Pan, and Post COVID-19 periods. HD, hospital data; CNIC, Chinese National Influenza Center. ns indicates no significant; ** indicates p<0.01; ****indicates p<0.0001.

Studies have suggested that the COVID-19 pandemic has influenced influenza transmission patterns. To explore this hypothesis, we analyzed influenza data from HD and CNIC, categorizing the periods based on COVID-19 control measures into pre-pandemic (Pre, 2019), pandemic (Pan, 2020–2022), and post-pandemic (Post, 2023–2024) phases. Our analysis revealed a significant decrease in the cumulative positivity rates of Flu A and Flu B during the Pan period in both HD and CNIC datasets (**Figures 5A, B**). Specifically, the positivity rates of Flu A in both HD and CNIC declined markedly during the Pan phase. In HD, Flu B positivity rates were lower in the Pan period compared to the Pre and Post phases. However, in the CNIC dataset, Flu B positivity during the Pan phase was higher than in the Post phase (**Figure 5B**).

Further analysis of the average weekly positivity rates indicated that, in HD, both Flu A and Flu B had significantly lower values during the Pan period compared to the Pre and Post periods. In the CNIC dataset, Flu A followed a similar trend, while Flu B showed no significant difference between the Pan and Post periods. Notably, in both HD and CNIC datasets, Flu B positivity rates were significantly higher in the Pre phase compared to the Post phase (**Figure 5C**).

Examining the proportions of Flu A and Flu B among influenza-positive cases, we found that in HD, Flu A dominated during the Pre, Pan, and Post periods, consistently accounting for over 80% of cases. In contrast, within the CNIC dataset, although Flu A had a higher proportion during the Pre and Post phases, it remained below 80%. Notably, during the Pan period, Flu B became the predominant pathogen, comprising 58% of influenza-positive cases (**Figure 5D**).

## Discussion

In this study, we analyzed the trends in Flu A and Flu B positivity rates from 2019 to 2024 at Sichuan Jinxin Xinan Women and Children Hospital and compared them with national surveillance data from the CNIC. Our findings revealed significant heterogeneity between local and national datasets in terms of influenza subtype distribution, seasonal patterns, and the impact of the COVID-19 pandemic. Notably, the suppression of influenza activity during the pandemic period was more pronounced for Flu A than for Flu B, with Flu B emerging as the dominant subtype in the CNIC dataset. Additionally, we observed a resurgence of influenza positivity rates in 2023, underscoring the need for strengthened post-pandemic surveillance and vaccination strategies. These findings highlight the importance of integrating both local and national data to obtain a comprehensive understanding of influenza dynamics and inform targeted public health interventions.

We found that both HD and CNIC data indicated that Flu A was the predominant type in 2019, with an average positivity rate exceeding 10%. This finding is consistent with results from multiple studies. A study in Yichang, a subtropical city in China, found an overall influenza virus positive rate of 16.6% among 8693 ILI cases, with a higher positive rate for Flu A (10.6%) than Flu B (5.9%) (Zhu et al., 2020). Another study in China, focusing on the period 2014-2018, reported an overall positive rate of 17.2% among 1,890,084 ILI cases, with Flu A detected in 62% of cases and Flu B in 38% (Zhu et al., 2023). In Cameroon, a study spanning from 2009 to 2018 indicated an influenza virus positivity rate of 24.0% among 11,816 participants with ILI (Monamele et al., 2020). These results suggest that before the COVID-19 pandemic, Flu A was the dominant strain of influenza.

The outbreak of the COVID-19 pandemic led to significant changes in influenza transmission patterns. A study reported that during the 2020–2021 influenza season, the global incidence of Flu A and B declined dramatically to just 0.0015 and 0.0028 times pre-pandemic levels (Lampejo, 2022). In the United States, reported influenza cases dropped sharply to only 1,899 during the 2020–2021 season, compared to millions of cases in previous years (Shaghaghi et al., 2021). Time-series analyses also indicate changes in influenza prevalence in China during the pandemic. One study found that during the 2020–2021 influenza season, the proportion of influenza-positive samples in southern China fell to 0.7%, whereas in previous seasons, this proportion ranged from 11.8% to 21.1% (Cao et al., 2023). From February 2020 to January 2021, the influenza positivity rate in China dropped to 0.2%, representing a significant decline compared to the same period in 2019 (Lei et al., 2020). The Chinese government established a Joint Prevention and Control Mechanism on January 2020, marking the beginning of its comprehensive response to the COVID-19 outbreak. This mechanism facilitated the rapid implementation of NPIs, including travel restrictions, quarantine measures, and public health campaigns aimed at educating the population about the virus (Deng and Grépin, 2024). On January 2023, China officially transitioned from a Class A to a Class B infectious disease management approach, signaling the end of the dynamic zero-COVID policy (Ge, 2023). This is why we defined 2019 as the Pre period, 2020–2022 as the Pan period, and 2023–2024 as the Post period in our study. In our study, HD results showed that during the COVID-19 pandemic, the influenza positivity rate dropped from approximately 25% before the outbreak to below 10%. After the pandemic, it rebounded to pre-pandemic levels. Similar findings were also observed in the CNIC data. These changes may be attributed to the stringent public health measures implemented to control the spread of COVID-19, such as lockdowns, social distancing, and mask mandates, which inadvertently reduced the transmission of influenza viruses. These non-pharmaceutical interventions significantly lowered influenza incidence during the 2020–2021 flu season (Chan et al., 2020; Itaya et al., 2020).

An important finding in our study is that, compared to Flu A, the COVID-19 pandemic appeared to have a lesser impact on Flu B. Our results show that, in 2021, Flu B was the dominant influenza type in both HD and CNIC data. While the limited sample size in HD might raise concerns about the reliability of the results, the CNIC dataset, which included over 400,000 tested samples, showed a similar pattern, with Flu B accounting for 99.87% of positive cases. This phenomenon had not been observed in previous studies. In the comparative analysis of the Pre, Pan, and Post periods, we also found that in the Pan period, Flu B accounted for more than 50% in the CNIC data, and in the Post period, the average positive rate for Flu B was slightly lower (though not statistically significant). During the COVID-19 pandemic, we observed that Influenza B (Flu B) replaced Influenza A (Flu A) as the predominant type of influenza. We speculate that this shift may be attributed to the following reasons:

The COVID-19 pandemic led to a significant decline in Flu A cases, with reports from many regions indicating that A/H1N1 and A/H3N2 strains were nearly eliminated. In contrast, Influenza B—particularly the Victoria lineage—persisted and even became dominant (Chang et al., 2023; Zhou et al., 2023); Compared to Influenza B, preventive measures such as social distancing and mask-wearing may have been more effective in reducing the transmission of Influenza A, allowing Influenza B to thrive (Wan and Zhang, 2022); The COVID-19 pandemic significantly altered the competitive dynamics of influenza viruses. As the prevalence of Flu A declined, Flu B filled the epidemiological niche and became increasingly prominent in the influenza landscape (Zeng et al., 2024).

Additionally, our study revealed discrepancies between HD and CNIC data. For example, an analysis of the weekly average Flu A positivity rate in the most recent year (2024) showed that the HD Flu A positivity rate was significantly higher than that of CNIC (p = 0.0008). Similar differences can also be observed in the figures from our study, highlighting the necessity of monitoring influenza trends at both the regional level and within individual centers. Furthermore, a comparative analysis between regional data and national influenza center data is crucial for understanding the characteristics of influenza epidemics and optimizing prevention and control strategies.

This study has several limitations that warrant further discussion. First, our dataset is derived from a single tertiary maternal and pediatric hospital in Chengdu, which may introduce selection bias. The patient population is primarily composed of children and women of childbearing age, who may have different healthcare-seeking behaviors, immune status, and vaccination coverage compared to the general population. Consequently, our findings may not be fully representative of the broader community, limiting the generalizability of the results. In our previous work on respiratory pathogen epidemiology in this hospital, we observed similar demographic limitations, emphasizing the need for caution when extrapolating hospital-based findings to population-level inferences (Li et al., 2025). Second, while national data from the Chinese National Influenza Center (CNIC) was used for comparison, a lack of transparency regarding critical surveillance parameters—such as catchment population size, geographic coverage, case definition, and laboratory protocols—limits our ability to draw direct comparisons between the HD and CNIC datasets. These system-level differences could contribute to the observed discrepancies in influenza subtype prevalence and seasonality. Third, vaccination coverage was not captured in the HD dataset, and reliable population-level estimates were not available. This presents a critical gap, as influenza vaccination is known to influence both individual susceptibility and transmission dynamics (Alexander et al., 2004). Without such data, we cannot assess whether the observed patterns were influenced by differential vaccine uptake, particularly in vulnerable populations such as children or the elderly. Fourth, changes in healthcare-seeking behavior during and after the COVID-19 pandemic may have influenced testing patterns and positivity rates. For example, reduced outpatient visits during the pandemic may have led to under-detection of mild influenza cases, while heightened awareness of respiratory symptoms in the post-pandemic period may have led to increased testing (Li et al., 2024). These shifts could introduce temporal bias in surveillance data. Fifth, our study focused exclusively on influenza positivity rates and did not include clinical outcomes such as hospitalization rates, ICU admission, or disease severity. These indicators would provide important context regarding the public health burden of influenza across different time periods and viral subtypes (Caini et al., 2018). Lastly, environmental and behavioral changes due to non-pharmaceutical interventions (NPIs) varied significantly across regions and time, potentially influencing the transmission dynamics of different influenza subtypes in ways not fully captured by surveillance data. Future studies should incorporate multi-center data with diverse population profiles, standardized surveillance protocols, individual-level clinical and vaccination data, and longitudinal follow-up to improve the accuracy, comparability, and public health relevance of influenza epidemiological assessments. Addressing these limitations will be crucial for optimizing influenza control strategies and improving our preparedness for future respiratory virus outbreaks.

In conclusion, our study reveals significant temporal variations in influenza activity, with notable differences in subtype distribution and seasonal trends between hospital-based and national surveillance data. The impact of COVID-19-related non-pharmaceutical interventions was more pronounced on Flu A than Flu B, highlighting the differential sensitivity of influenza subtypes to public health measures. These findings underscore the importance of integrating multiple surveillance sources for a comprehensive understanding of influenza dynamics. Strengthening vaccination coverage and adaptive public health strategies will be essential for mitigating the influenza burden in the post-pandemic era.

## Data Availability

The raw data supporting the conclusions of this article will be made available by the authors, without undue reservation.
